# Blood-Based Biomarkers for Eosinophilic Esophagitis and Concomitant Atopic Diseases: A Look into the Potential of Extracellular Vesicles

**DOI:** 10.3390/ijms24043669

**Published:** 2023-02-11

**Authors:** Elena Grueso-Navarro, Pilar Navarro, Emilio J. Laserna-Mendieta, Alfredo J. Lucendo, Laura Arias-González

**Affiliations:** 1 Department of Gastroenterology, Hospital General de Tomelloso, Tomelloso, 13700 Ciudad Real, Spain; 2 Instituto de Investigación Sanitaria de Castilla-La Mancha (IDISCAM), 45004 Toledo, Spain; 3 Laboratory Medicine Department, Hospital Universitario de La Princesa, 28006 Madrid, Spain; 4 Instituto de Investigación Sanitaria Princesa (IIS-IP), 28006 Madrid, Spain; 5 Centro de Investigación Biomédica en Red de Enfermedades Hepáticas y Digestivas (CIBERehd), Instituto de Salud Carlos III, 28006 Madrid, Spain

**Keywords:** eosinophilic esophagitis, atopic dermatitis, bronchial asthma, Th2-inflammation, eosinophils, extracellular vesicles, serum, plasma, biomarker

## Abstract

Eosinophilic esophagitis (EoE) is a chronic, Th2-inflammatory disease of the esophagus that can severely affect food intake. Currently, diagnosis and assessing response to treatment of EoE is highly invasive and requires endoscopy with esophageal biopsies. Finding non-invasive and accurate biomarkers is important for improving patient well-being. Unfortunately, EoE is usually accompanied by other atopies, which make it difficult to identify specific biomarkers. Providing an update of circulating EoE biomarkers and concomitant atopies is therefore timely. This review summarizes the current knowledge in EoE blood biomarkers and two of its most common comorbidities, bronchial asthma (BA) and atopic dermatitis (AD), focusing on dysregulated proteins, metabolites, and RNAs. It also revises the current knowledge on extracellular vesicles (EVs) as non-invasive biomarkers for BA and AD, and concludes with the potential use of EVs as biomarkers in EoE.

## 1. Introduction

Eosinophilic esophagitis (EoE) is a chronic disease characterized clinically by symptoms referred to as esophageal dysfunction, and histologically by eosinophil-predominant inflammation of this organ [1]. EoE is mainly driven by food-antigens that trigger T-helper 2 (Th2) local immune response [2,3], and is considered as a particular form of food allergy that frequently appears in patients who suffer from other Th2-associated atopies [4]. EoE is recognized as the leading cause of chronic dysphagia in children and young adults, and the second cause of chronic esophagitis after gastroesophageal reflux disease (GERD) [5]; and there has been a constant rise in its incidence, with currently 34.4 new cases/100,000 subjects per year [6]. Although progress in management of the disease has significantly reduced diagnostic delay over recent years [7], endoscopy with esophageal biopsies still remains the only reliable method for EoE diagnosis, monitoring disease activity and assessing the response to treatment. Thus, finding of a non-invasive, accurate and specific biomarker is a key goal, and an area of great potential in the research of this disease. Despite the efforts being made however, minimally invasive biomarkers are not yet being applied to routine clinical practice [8].

One of the main obstacles in the search for non-invasive biomarkers is the lack of specificity. This is complicated greatly by the frequent concomitance of EoE with several atopic conditions, including atopic dermatitis (AD), bronchial asthma (BA), IgE-mediated food allergies, and allergic rhinitis, all significantly more common in EoE patients than in the general population [4,9,10]. In fact, EoE has been proposed as a late manifestation of the “atopic march”, a natural concatenation of atopic disorders over time [11], with BA and AD being among the most common comorbidities of EoE [10]. Despite this strong association, only a small proportion of studies have included atopic controls [12] as shown in Table 1, thus hampering the proper identification of suitable biomarkers.

Extracellular vesicles (EVs) are a tremendously heterogeneous group of membrane-limited entities, of nanometric size, released into the extracellular space by virtually all cell types and cellular organisms [21]. Although initially conceived as cell ‘debris’ or cellular waste, evidence of their relevance in both physiological and pathological scenarios, mainly defined by their role in intercellular communication, has driven forward research since the early 2000s [22]. In allergy, EVs intervene actively in different aspects of its pathophysiology, from induction of inflammation by activation of allergen-specific T cells, to the contribution of sustained chronic inflammation and development of fibrosis [23]. This is possible due to an orchestrated mechanism in which EVs are released and uptake by cells of the inflammatory microenvironment, whose effects are greatly determined by their content (i.e., inflammatory cytokines, enzymes, miRNAs, etc.) [23,24,25]. Moreover, EVs have arisen as the most promising source of biomarkers for several reasons. First, EVs are abundant in many bodily fluids such as blood, urine, saliva, ascites fluid, pleural effusion, breast milk, and cerebrospinal and bronchoalveolar lavage fluid [26]. Moreover, EVs’ intraluminal and extraluminal cargo (proteins, nucleic acids, lipids, and metabolites) are functional [27], and correlate well with their parental cell. They inform the cell identity and its biological status, thus acting as a peripheral representation of a pathological process. Finally, EVs have a significant advantage over other serum-biomarkers now in that their conformation extends the stability of their cargo such as RNAs and proteins, protecting them from catabolic enzymes in circulation. Despite this, the nanometric size of EVs, and the complex composition of biofluids as blood are technically challenging and need to be overcome in order to accelerate their use in diagnostics [28].

This review updates current knowledge relating to potential non-invasive blood-based biomarkers described for EoE and two of its most common comorbidities: BA and AD, with the aim of identifying the common unspecific biomarkers. We also summarize state of the art use of EVs as circulating biomarkers for asthma and atopic dermatitis and speculate about the advantage of using EVs as non-invasive biomarkers for diagnosis and the management of EoE.

## 2. Methods

We searched the PubMed library, using the following individual and combined key words: eosinophilic esophagitis; atopic dermatitis; eczema; bronchial asthma; extracellular vesicles; exosomes; blood; circulating; serum; plasma; biomarker. Reference lists in the articles obtained were also searched in order to identify other potential sources of information. We included studies describing circulating biomarkers if performed in human samples, study subjects suffering from EoE, BA, or AD, together with other comorbidities. The results were limited to studies published and written in English and carried out on humans or human samples.

## 3. Current Knowledge of Circulating Biomarkers

EoE, BA, and AD are all chronic inflammatory diseases characterized by Th2 immune responses. Upon exposure to an allergen, sensitization occurs in the epithelial barrier of the esophagus (EoE), airways (BA), and skin (AD), the epithelial integrity of which is disrupted as a result of defects in cell–cell contacts [26,27]. Sensitized epithelial cells then orchestrate the immunological response by releasing alarmins (IL-25, IL-33, and TSLP) responsible for the polarization of CD4+ T cells towards Th2 phenotype [29,30,31]. Cytokines released by Th2 lymphocytes (IL-4, IL-5, and IL-13) stimulate, among others things, the proliferation of eosinophils that are subsequently recruited to the inflammatory foci from circulation, causing the eosinophilia characteristic of EoE, BA, and AD [32,33,34,35]. The cytotoxic nature of eosinophil-granule proteins released locally then promotes skin lesions and pruritus in AD [36]. As a result, remodeling and hyperresponsiveness of the airways in BA [37], and esophageal dysmotility and fibrosis in EoE occurs [38]. Unsurprisingly, there is a significant overlap in the molecular mechanisms of these three diseases that share up to 18 dysregulated genes [39]. However, EoE, BA, and AD are good examples of diverse eosinophilic disorders with different tissues being affected, mediation of serum IgE, and systemic/local inflammation. AD and BA have increased serum total IgE and allergen-specific IgE levels in common [32,34], therefore skin prick testing (SPT) is a suitable tool for diagnosis its use in EoE is limited however as IgE is not required for its pathogenesis [40]. In addition, AD is considered a systemic disease [41], while EoE and BA, with the exception of an endotype of BA [42], are characterized by local inflammation in the esophagus and the airways, respectively. 

Since the pathophysiology of EoE, BA, and AD is very similar, it is likely that unspecific biomarkers could be found when using samples such as blood the most common tissue used when looking for non-invasive biomarkers and therefore, will be the focus of this review. It should be noted that overall biomarkers for EoE [43], BA [44], and AD [45] have been reviewed recently. As counts of circulating eosinophil and serum IgE levels have been widely employed in EoE, BA, and AD, with inconclusive results, they will not be reviewed here.

### 3.1. Bronchial Asthma (BA)

#### 3.1.1. Proteins

The serum levels of periostin, a matricellular protein involved in eosinophilic inflammation and airway remodeling [46], are thought to be a promising biomarker for Th2-eosinophilic asthma for two main reasons: firstly, it correlates well with its expression in the airways, and secondly, it remains relatively stable in blood [47]. In fact, serum periostin can predict airway’s eosinophilia alone [48] and thus serves well as a diagnostic and predictive biomarker. Furthermore, its expression is markedly increased in children from 2 to 11 years old, compared to adults [49], despite exhibiting a limited diagnostic ability in children with severe asthma [50]. The diagnostic potential of osteopontin (OPN), another matricellular protein, was first suggested by Samitas et al., who observed increased levels of serum OPN in asthmatic patients compared to healthy controls [51]. Although such overexpression was later validated [52], a meta-analysis including 9 studies, in which 7 of them used serum or plasma OPN, concluded that there was no association between circulating OPN and asthma and was not useful for diagnostics or to reflect severity [53].

Upon activation, eosinophils release their granule proteins (ECP, EDN, EPX, MBP-1, and CLC/Gal-10), which are cytotoxic proteins involved in eosinophil-inflammation, tissue remodeling, and serve as indirect markers of inflammation [54]. In BA, serum eosinophil cationic protein (ECP), which appears overexpressed in the serum of both adult and pediatric patients, has been the most widely studied granule protein [55,56]. Although its utility as a diagnostic biomarker is limited, its correlation with disease severity and response to treatment exhibited greater potential [57,58]. The lesser studied eosinophil-derived neurotoxin (EDN) has arisen as a promising clinical biomarker, informative of diagnosis, severity, and treatment monitoring [59]; more importantly, EDN is very stable in blood samples, thus increasing its analytical reliability [60]. Blood C-reactive protein (CRP) has also been proposed as a marker of airway inflammation, but in a very limited cohort of non-smoker asthmatic patients without such additional complications as cardiovascular-related diseases, hyperlipidemia, chronic obstructive pulmonary disease (COPD), or infection [61].

Several proteins involved in tissue damage and remodeling appear dysregulated in the blood of asthmatic patients. In a meta-analysis of 17 studies, the extracellular matrix glycoprotein YKL-40, was found upregulated in asthmatic patients compared to healthy controls, and correlated with disease severity and acute exacerbation of the disease, regardless of COPD and related syndromes [62]. Moreover, metalloproteinase-9 (MMP-9) and dipeptidyl peptidase-4 (DPP-4), involved in extracellular matrix remodeling, are both elevated in the serum of BA patients, and their expression, related to disease severity, is reduced after treatment with corticosteroids [58,63]. In fact, corticosteroid refractoriness, an important issue in the management of BA, could be identified by the downregulation of mucin-1 (MUC-1) in circulating neutrophils, a characteristic of patients with uncontrolled severe asthma [64]. Hur et al. explored the ability of several serum cytokines, periostin, EDN, calprotectin (S100A9), and folliculin to distinguish BA phenotypes, and concluded that increased serum concentrations of folliculin and calprotectin discriminated paucigranulocytic and neutrophilic phenotypes, respectively [65].

Serum levels of several cytokines, including Th2-cytokines such as interleukin (IL)-25, IL-4, IL-5, IL-13, and IL-33, are elevated in asthmatic adults and children compared to controls [66]. Increased serum levels of the chemokine CCL26/eotaxin-3 could differentiate moderate-to-severe asthma from healthy controls [67], and recent findings point out the ability of CCL17/TARC to predict type-2 eosinophilic asthma [68]. Upregulated IL-8 and TNFα were detected in acute attacks of asthma [69], and IL-1β is an indicator for the risk of pediatric allergic asthma [70].

#### 3.1.2. Metabolites

A number of studies have identified metabolite dysregulation as useful for diagnosis, phenotyping, assessing of severity, and response to treatment [71]. However, the lack of analytical standardization sabotages replicability [72]. Therefore, only those metabolites that appear dysregulated in at least two different studies using plasma or serum samples from asthmatic patients were considered. Only linoleic acid levels resulted consistently significant in active asthma [73,74], although correlation with severity was not found [74]. In contrast, levels of glycerophosphocoline and L-valine were downregulated [75,76,77]. However, the majority of selected metabolites exhibited opposing results, appearing down- and upregulated in asthma indistinctly, which could be explained by the variability in the origin (whole blood, plasma, or serum) or population type (adult or children) of where/from whom samples were collected. The metabolites include arachidonic acid [75,77], succinate [75,78], palmitic acid [75,79], xanthine [74,75], taurine [74,76], bilirubin [74,75], arginine [75,80], histidine [80,81], and glucose [73,80].

#### 3.1.3. RNA

Changes in gene expression overall suggest the potential of circulating RNAs as BA biomarkers [82,83,84,85], with miRNAs being the most commonly studied. In 2016, Panganiban et al. described that the differential expression of 30 miRNAs in plasma distinguished a cohort of patients with allergic rhinitis and 2 subtypes of asthma with high or low peripheral eosinophil counts from healthy controls [85]. In total, 11 miRNAs were upregulated (miR-125b, miR-126, miR-21, miR-16, miR-223, miR-148a, miR-146a, and let-7b/c/e) and 5 downregulated (miR-1, miR-299-5p, miR-570, miR-106a and miR-155) exclusively in asthma samples [85]. A further analysis compiling different studies concluded that the combination of miR-185-5p, miR-155, miR-21, miR-320, miR-1246, miR-144-5p, miR-1165-3p, and let-7a potentially served as a diagnostic biomarker for asthma [86]. In line with previous reports, Kyyaly et al. proposed a panel of circulating miRNAs with diagnostic (upregulated miR-126, miR-155, miR-21, miR-125b, miR146a, and miR-98, and downregulation of let-7 family, miR-192, miR-15a, and miR-30a) and assessment of severity potential (upregulated miR-126, miR-155, miR-125b, and miR-1165-3p, and downregulated miR-1 and miR-19b) [84]. 

In some cases, a correlation between certain miRNAs and lncRNAs served as biomarkers of disease exacerbation. This is the case for ANRIL/miR125a or NEAT1/miR124 [87]. Other lncRNAs are informative for BA diagnosis in adults (RP11-401.2 and LNC-000127) [88], and children (CASC2, PTTG3P, lncRNA-H19) [89,90,91]. Among mRNAs, the upregulation of PTGDR2, a prostaglandin receptor involved in the chemotaxis of leukocytes, was found to be significantly upregulated in the blood of asthmatic patients [92].

### 3.2. Atopic Dermatitis (AD)

#### 3.2.1. Proteins

As eosinophils are active participants in the pathogenesis of AD, eosinophil granule-derived proteins are found in circulation, although their utility as biomarkers is under debate. ECP was postulated in the early 1990s as a severity biomarker [93,94], and EDN subsequently [95]. However, later studies in pediatric patients showed no significant association [96,97]. 

Several adhesion molecules appeared upregulated in skin biopsies of AD patients [98], and some have also been detected in circulation. Increased serum periostin has potential as a diagnostic and severity biomarker [99], and its levels even vary in response to therapy [99,100]. E-selectin and tissue remodeling matrix metalloproteinases (MMP-3/9/10/12) are also potentially useful for diagnosis [101,102]. 

In AD, Th2-chemokines such as CCL26/eotaxin-3 [103,104], CCL22/MDC [105], CCL18/PARC [100,106], CCL27/CTACK [107], and CCL17/TARC, have been more commonly found as blood biomarkers compared to Th2-cytokines [45], the latter of which is considered the most reliable biomarker for AD [66]. In fact, elevated serum TARC/CCL17 discriminated AD from healthy controls [102,108,109] and showed the best odds ratio (OR) when compared to eosinophil count, total IgE, serum IL-18, and lactate dehydrogenase (LDH) [110]. However, more extensive evidence promotes TARC’s utility as a biomarker of severity [111,112,113], and response to treatment [100]. Its specificity has been questioned however as it also appears upregulated in several skin diseases [45], with the exception of psoriasis [102]. A few cytokines with biomarker potential in AD include IL-13, IL-22 [100,109], and IL-18 [110,114]. Contradictory results relating to DPP-4 showed its upregulation in plasma of AD patients [115], while in circulating T-cells in AD, it exhibited a significant decrease in surface expression [116,117].

Other inflammatory molecules are: the soluble receptor IL-2 (sIL-2R), associated with severity [93,94] but with inconclusive results [102]; soluble CD23 (sCD23) [94]; the receptors of soluble Tumor Necrosis Factor (sTNFRI/II) [118]; C-reactive protein (CRP), which is upregulated in AD subjects compared to healthy controls and correlated with disease severity scores [119]; and lactate dehydrogenase (LDH), which is also elevated in AD [99,119] as a potential indicator of disease severity both in children [120], and adults [110]. Adipokines, involved in the integration of metabolism and immune function [121], have also been postulated as potential biomarkers. A significant decrease of serum resistin and adiponectin differentiated adults with AD from controls, and correlated with the SCORing Atopic Dermatitis (SCORAD) index [122]. Similarly, serum levels of YKL-40 appeared significantly upregulated in AD versus healthy controls, and correlated positively with the SCORAD index, thus, indicating its potential as a biomarker for severity [123]. Likewise, elevated serum levels of squamous cell carcinoma antigens 1 and 2 (SCCA1/2) have been described in AD and psoriatic patients [124].

#### 3.2.2. Metabolites

A distinct metabolic signature of AD has been most commonly studied using skin biopsies rather than in circulation [125], reflecting the stronger diagnostic power of skin compared to blood [126,127]. Existing studies in serum samples identified a distinct metabolic profile between AD and healthy controls involving dysregulation of phosphatidylcholine and acylcarnitine [128], and other metabolites and lipid mediators that are linked to the pro-inflammatory state of AD [129]. In plasma, Chiu et al. showed that a metabolic signature related to the metabolism of nitrogen and amino acids discriminated AD endotypes based on filaggrin mutations and IgE levels [130]. This was further evinced in pediatric patients, regarding elevated IgE endotypes [131]. In addition, metabolic signatures have identified therapeutic responders as shown for omalizumab and dupilumab treatments [132,133]. Only recently, the increased levels of vitamin D and A5 ligands in the plasma of AD patients has been postulated as a biomarker of AD severity [134].

#### 3.2.3. RNA

Cumulative evidence connects microRNAs (miRNAs) with the pathophysiology of AD and other skin disorders [135,136]. This has motivated the search for differential circulating miRNA-expression patterns in blood in order to seek a suitable biomarker. In an initial study involving pediatric subjects, serum levels of miR-203 and miR-484-5p were found to be significantly increased in children with AD compared to controls. However, miR-203 was upregulated exclusively in those patients who showed high serum IgE levels [118]. In addition, miR-155, known to be upregulated 4–6-fold in AD skin [137], also appeared significantly increased in the circulation of atopic children [138] and circulating CD4+ monocytes of children with AD [139].

A subsequent study performed with a small cohort of adult patients with AD, psoriasis, and healthy controls, showed that serum levels of miR146a, miR-203, and miR-205 had no discriminative ability for AD compared to controls; however, it exclusively identified significant downregulation of serum miR-125b in AD patients [140]. These results contradict the aforementioned miR-203 upregulation in AD [118]. A recent study carried out in pediatric patients suffering from AD found a significant increment in serum miR-146a expression, and a lower ratio of Th1/Th2 compared to controls [141]. Another study that selected miR-29b based on a miRNA microarray of skin biopsies, demonstrated its upregulated expression in the serum and correlation with the disease severity score [142].

One of the two studies to date that used plasma instead of serum as a source of circulating biomarkers for AD identified 25 differentially regulated miRNAs by high-throughput sequencing of samples from 700 subjects (including adult and pediatric patients, and healthy controls without history of atopies). Of those upregulated sequences, plasma miR-151a was significantly increased in AD when confirmed by RT-qPCR [143]. The second study, using plasma samples of children with AD, showed the dysregulation of 40 miRNAs and proposed the most differentially expressed miRNA, hsa-miR-194-5p, as a potential biomarker of AD, based on area under the receiver operating characteristic (ROC) analysis [144].

In addition to microRNAs, a significant increase of mRNAs encoding for pro-inflammatory cytokine IL-17 and retinoic acid receptor (ROR)γt in circulating Th17- CD4+ cells obtained from AD patients suggest their potential as a diagnostic biomarker for the disease [139].

### 3.3. Eosinophilic Esophagitis (EoE)

#### 3.3.1. Proteins

Periostin is markedly overexpressed in the esophagus of EoE patients [145], and represents a promising non-invasive biomarker. Slightly increased levels of serum periostin differentiated EoE patients from controls in correlation with high serum IL-13 [146]. However, this is, to our knowledge, the only study assessing serum periostin. Alternatively, given the disruption of the epithelial barrier and esophageal fibrosis in EoE, the use of autoantibodies against epithelial adhesion molecules has been hypothesized as serving as disease biomarkers. Indeed, antibodies against transmembrane desmoglein-3 (DSG3) and collagen XVII (NC16A) appeared to be increased in the serum of EoE patients, with a more prominent increase of NC16A [147].

ECP and EDN are the eosinophil granule proteins most commonly studied in circulating blood in the context of EoE. Although no utility of ECP or EDN as biomarkers was reported in a small number of studies [148,149], this evidence was overshadowed by others showing the upregulation of EDN [16,150,151] and ECP [150,152,153] in the blood of EoE patients compared to healthy controls. Consequently, treatment with mepolizumab led to a reduction in ECP and EDN levels [154], and topical corticosteroids [155] or diet restriction also reduced ECP levels [156] with variable results [157]. Despite the lack of differences in circulating MBP-1 initially reported [148], more recent works have shown a significant upregulation of MBP-1 as being helpful in discriminating EoE from healthy controls, and after treatment [158], and even in combination with CLC-GAL10 [153]. Strikingly, in contrast to common upregulation of granule proteins reported to date, Wright et al. observed lower levels of granule proteins in the serum of EoE patients that exhibited a marked degranulation within the tissue [149]. They postulated that circulating eosinophils in EoE might retain their granules, therefore suggesting that downregulation of serum EPX is a biomarker of EoE [149].

Cytokines, as mediators of inflammation, have been extensively studied as inflammatory disease biomarkers. The blood levels of cytokines: IL-4, IL-13, IL-5, IL-6, IL-12p70, CD40ligand, IL-1α, and IL-17 distinguished EoE from non-EoE patients [159], but such changes could not be validated in subsequent studies [66]. Among cytokines, IL-5 has raised the highest interest given its key role in the proliferation, maturation, and differentiation of eosinophils within the bone marrow [160]. However, many studies could not demonstrate differences in serum IL-5 [148,150,151,158,161]. Only Lu et al. [162] observed a significant increase of Th2 cytokines, specifically IL-10, in EoE patients, together with increased levels of absolute eosinophil counts and serum levels of TNFα and IL-12 cytokines. Similarly, despite the increased esophageal expression of eotaxin-3 [163], a chemokine important for eosinophil migration and tissue infiltration, differences in circulation have not been detected [150,153,158]. The exception is a study performed in pediatric patients, where eotaxin-3 was increased in plasma, along with increased absolute eosinophil counts and tissue eosinophilia [16].

#### 3.3.2. Metabolites

Just two studies highlighted dysregulated metabolites in circulation as being possibly useful for EoE diagnosis. This is the case with 15(S)-hydroxyeicosatetraenoic acid (15(S)-HETE), a metabolite detected in peripheral blood derived from arachidonate 15-lipoxygenase (ALOX15), which is upregulated in the esophagus of EoE patients, and also found to be increased by 2.4-fold in the serum of EoE patients [164,165]. A further study identified several urea cycle metabolites (dimethylarginine, putrescine and N-acetylputrescine) as potential biomarkers for EoE diagnosis in children [166].

#### 3.3.3. RNA

Lu et al. tested for the first time the potential of circulating microRNAs as a reflection of the RNA expression profile in tissue. They found that increased levels of miR-146a, miR-146b, and miR-223 significantly differentiated between healthy controls and EoE patients, while downregulation of miR-146a and miR-233 indicated EoE remission induced by glucocorticoid therapy [167]. Likewise, significant upregulation of serum miR-21 30-fold in EoE correlated with an increased expression 40-fold in the esophageal tissue [19]. Unfortunately, there is still little evidence of circulating microRNAs in EoE, and other authors failed when they tried to detect them in the serum [168]. Differences in mRNAs have also been explored as potential circulating biomarkers for EoE [169,170,171]. The presence of IgE receptor I (FcεRI) mRNAs was found to be significantly reduced in the blood of EoE patients compared to GERD and healthy controls [169], a finding that aligns with the fact that EoE is a non-IgE-mediated allergy [40]. Similarly, IL-15R mRNAs were less abundant in the blood of EoE compared to GERD patients [169]. Despite IL-15 expression being induced in the esophagus of active EoE [172], the authors hypothesized that the reduction in circulating lymphocytes with IL-15 receptors was due to their preferential recruitment to the tissue. On the other hand, upregulation of mRNAs encoding for eosinophil surface molecules such as CD101, CXCR6, and CD274 (PDL1) served to discriminate EoE from GERD patients [170,171].

**Figure 1:**
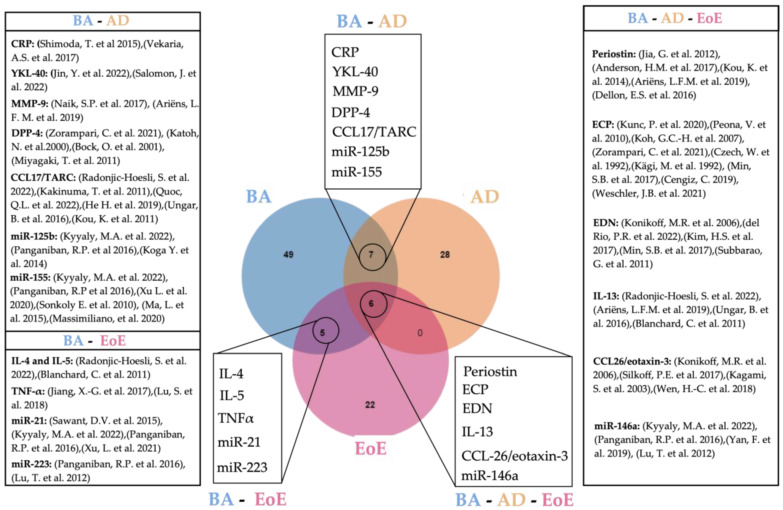
Venn diagram showing overlapping circulating biomarkers for BA, AD, and EoE [16,19,47,48,49,54,55,56,57,58,59,60,61,62,65,66,67,68,83,84,85,92,93,94,99,100,101,102,103,107,108,114,118,122,123,136,137,138,139,144,148,150,151,152,153,154,157,160,165]. Created with Jvenn diagram viewer [173]. BA: bronchial asthma, AD: atopic dermatitis, EoE: eosinophilic esophagitis.

Common circulating biomarkers for either BA and AD, BA and EoE, AD and EoE, or BA, AD, and EoE are shown in Figure 1.

## 4. Extracellular Vesicles (EVs) as Circulating Biomarkers

### 4.1. EVs and the Immune System

The term extracellular vesicle was coined in 2011 [174] to define any non-replicating extracellular entities delimited by a lipidic membrane that are released by cells. Although the classification is complex and continues to grow as the field develops [22], EVs can be generally divided in two broad categories based on their biogenesis: endosomal origin (or exosomes), and plasma-membrane (or ectosomes) [25]. Regardless of their biogenesis, EVs perform fundamental functions in homeostasis and pathological processes that range from the removal/recycling of unnecessary molecules from the cell to the delivery of intercellular signals. The biogenesis of EVs, as well as the identity and state of the parental cell, largely contribute to defining their cargo and, consequently, their function [175]. 

The ability of EVs to mediate intercellular communication is of enormous relevance in immune signaling. In inflammation and innate immunity, EVs exert pro- and anti-inflammatory functions mediated by their load of bioactive lipids (i.e., arachidonic acid) [176]; cytokines [177]; damage-associated molecular patterns (DAMPs) [178]; pro-inflammatory microRNAs [179]; or soluble mediators (i.e., C-reactive protein) [180]. Activation of immune responses upon microbial or allergen intrusion also involves EVs, as they carry allergens and microbial-associated molecular patterns [25]. In addition, a fundamental function in adaptive immunity, antigen presentation, can be mediated by MHC-loaded EVs, contributing to a more sophisticated regulation of immunity [181,182]. Viewing the published data, it is logical to think that EVs can be utilized as biomarkers, especially in the context of immune mediated disorders, where EVs seem to have ubiquitous roles. We will now examine the data suggesting the utility of circulating EVs as biomarkers of AS and AD.

### 4.2. Circulating EVs as Biomarkers in BA

One of the first hints indicating an association between asthma and EVs was the increment of such particles in the circulation of asthmatics [183]. Duarte et al. found elevated levels of platelet microparticles in the circulation of a group of 20 asthmatics under corticosteroid treatment compared to 15 healthy volunteers, and further studies showed similar upregulation induced by pollution [183,184,185]. Nonetheless, elevated EV levels do not correspond specifically to asthma, as was observed in asthmatics and non-asthmatics from a cohort of type-1 allergic patients with rhinoconjunctivitis [186].

Identifying significant differences in the EV-cargo is one of the main hopes when looking for EV biomarkers, and EV-miRNAs are among the most commonly studied molecules in this regard. Increased levels of serum EV miR-126 in allergic asthmatic patients has suggested its potential as a diagnostic biomarker [187]. MiRNA-125b in serum EVs appeared upregulated in patients with different levels of asthma severity, and was even able to discriminate mild-bronchial asthma from healthy controls [188]. Consistently, upregulation of EV-miR-125b correlated with increased CRP and IgE serum levels, which together with downregulated EV- miR-124, miR-133, and miR-130, could differentiate subjects with severe asthma under corticosteroid treatment from healthy controls [189]. The let-7 family of microRNA is dysregulated in bronchial asthma [84]. Accordingly, Zheng et al. observed that increased let-7i-5p levels in plasma EVs correlated with patient exposure to fine particulate matter in a cohort of asthmatic children and, in combination with serum IgE, exhibited a greater diagnostic performance [185]. Plasma EVs significantly enriched in miR-223 and miR-21 also differentiated moderate asthmatic patients from healthy controls [190]. The only study, to our knowledge, that employed RNAseq in circulating human EVs for plasma did not find overall changes in the miRNA content; however, an insufficient cohort size could partly explain these results. Nonetheless, the authors identified the upregulation of miR-122-5p in all groups of eosinophilic asthma compared to controls [191].

Although less explored, proteins in EVs harbor potential biomarkers for defining asthma endotypes, as hypothesized by Suzuki et al. [192]. Without purification of EVs, the proteome of plasma samples from COPD or patients with severe asthma showed different profiles differentiating each disease and asthma endotypes, significantly enriched in extracellular vesicle markers, and thus suggesting an association between EVs and protein groups [192]. Similarly, a pattern of upregulated (TNFα, IL-4, IL-5, IL-6, IL-17F, CCL2, and CCL17/TARC) and downregulated (IL-11, IL-27, and CCL20) EV-associated cytokines discriminated healthy controls from allergic patients, including asthmatics [186].

### 4.3. Circulating EVs as Biomarkers in AD

The pathogenesis of AD has been associated with the colonization of the skin by different microbes, one of the best characterized being *Staphylococcus aureus* [193]. The fact that bacteria can release EVs [194] has sustained the research around microbe-derived EVs as potential pathogenic effectors and biomarkers. A metagenomic analysis on serum and urine EVs from AD and healthy donors identified high homogeneity between both type of biofluids, and significant downregulation of several lactic acid bacteria genera in comparison to controls in urine EVs [195]. A similar study including a greater number of healthy controls, and using only serum EVs, identified *Escherichia*–*Shigella* and *Enterococcus* as upregulated in AD [196], which suggests great potential for microbial EVs as biomarkers for AD. 

EVs derived from immune cells can reflect their origin and the activation state of the donor cell by their content and surface marker [25]. Based on this premise, Ryutaro Oba et al. identified several EV-subsets from T cells: CD3+CD4+ EVs for CD4+ T cells, CD3+CD8+ EVs for CD8+ T cells, and CD3+HLA-DR+ EVs for Th1-type T cells [197]. Interestingly, differential distribution of such EV subsets could discriminate AD from healthy adults as CD3+CD4+ EVs were significantly upregulated in AD, while CD3+HLA-DR+ EVs were downregulated. However, when AD was compared to other inflammatory diseases such as osteoarthritis or rheumatoid arthritis, no differences were found in the aforementioned EV-subsets. EVs derived from mast cells have been identified in the serum of both AD and non-atopic controls, although a significant overexpression of the miR103a-3p characterized EVs from AD patients [198]. RNAseq of plasma derived EVs from pediatric AD patients revealed 10 differentially regulated genes, among which the transfer RNA Fragment *tRF-28-QSZ34KRQ590K* (tRFs are a novel class of non-coding RNAs with regulatory roles [199]) was significantly downregulated in AD [200]. To date, a single study has characterized the proteomic differences in serum EVs in the context of AD [201]. Over a thousand proteins were identified, of which 19 were unique to AD-EVs, with overrepresented functions linked to pro-inflammatory cytokine production such as platelet activation or Rap1 signaling [202].

**Figure 2:**
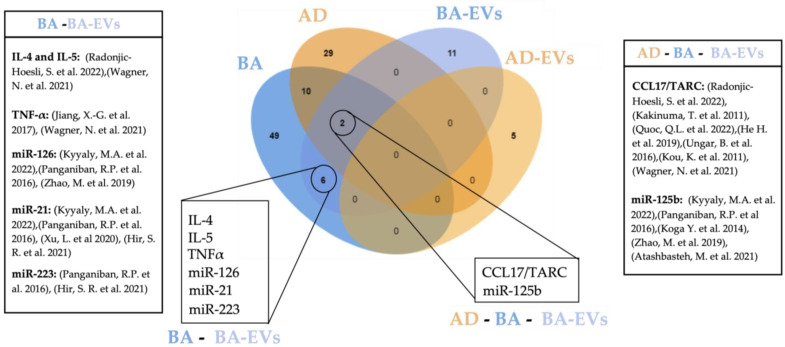
Venn diagram showing overlapping circulating biomarkers for BA, AD, BA-EVs, and AD-EVs [65,66,67,68,83,84,85,101,107,108,138,184,185,186,187,188]. Created with Jvenn diagram viewer [173]. BA: bronchial asthma, BA-EVs: bronchial asthma extracellular vesicles, AD: atopic dermatitis, AD-EVs: atopic dermatitis extracellular vesicles.

Common biomarkers between BA, BA-EVs, AD, and AD-EVs are shown in Figure 2.

### 4.4. Potential of EVs as Biomarker of EoE

Although the role of EVs remains unexplored in EoE, a number of studies have collected evidence of the functional relationship of EVs, with two areas closely linked to EoE: allergy and inflammation [203], and esophageal disorders (mostly Barrett’s esophagus) [204]. Development of Barrett’s esophagus is often preceded by sustained GERD, which exhibits overlapping symptoms with EoE but a different pathogenesis [205]. Preliminary work by Uemura et al. showed the diagnostic potential of EV-miRNAs in a rat model of GERD, in which serum EVs were withdrawn from male rats at different stages after the development of reflux esophagitis (acute, sub-acute, and chronic phases) [206]. By microarray analysis of the EVs, miR-223-3p and miR-29-3p were identified as differentially expressed among the different phases. Interestingly, the upregulation of circulating miR-223 has previously been found upregulated in EoE patients compared to healthy controls [167].

The highly conserved mechanisms of autophagy coordinate the recycling and degradation of cellular material to maintain protein homeostasis, activated in response to certain stress stimuli such as inflammation [207]. In line with this, a study employing human biopsies and murine models of EoE demonstrated that inflammatory stimuli mediated by IL-13 and TNFα cytokines upregulated autophagy in esophageal epithelial cells, causing the accumulation of autophagic vesicles (AVs) with distinct cargo between EoE and control samples [208]. Autophagy and EVs are tightly related, due to the confluence in their mechanisms of biogenesis and functionality, since the autophagy–EV crosstalk is relevant in homeostatic and pathologic scenarios [209]. Moreover, the release of extracellular vesicles and particles with autophagic cargo controlled by the secretory autophagy pathway has been described [210,211]. Future studies might consider exploring the release of vesicles to the extracellular milieu, that potentially contain autophagy-derived material, as being useful as biomarkers in EoE. 

The total of specific markers that define the tissue origin of EVs is still largely unknown. According to Li et al., about 0.2% of EVs in circulation belong to different tissues, including EVs that derive from the esophagus, as several specific genes were identified in plasma-EVs [212]. However, the vast majority of circulating EVs are generated by blood cells (i.e., monocytes, lymphocytes, platelets, etc.), suggesting that immune cell-derived EVs are the most promising as a source of circulating biomarkers. In fact, every immune cell with a role in inflammation can secrete EVs [25]. This is the case for eosinophils [213]. Studies employing EVs derived from circulating eosinophils demonstrated that, upon cytokine stimulation (IFNγ, eotaxin-1, or ΤΝFα), these cells increase the release of EVs carrying several eosinophil-granule proteins, which are more prominent in eosinophils from asthmatics than in healthy controls. Moreover, eosinophil-EVs participate in tissue remodeling and cell migration, indicating their active role in disease pathophysiology [214]. Despite the potential of eosinophil-derived EVs as biomarkers for eosinophilic disorders, the characterization of these EVs is currently limited and studies reporting the detection of eosinophil-EVs in biofluids are lacking. 

Changes in the microbiota of the mucosa have been linked to the initiation and maintenance of inflammation. The flora of the healthy esophagus, commonly colonized by the genus *Streptococci* [215], is imbalanced in EoE esophagi towards an enrichment in *Neisseria*, *Corynebacterium* [216], and *Haemophilus* [217]; and downregulation of *Phorphyromonas* [218]. Both commensal and pathogenic bacteria can secrete bacterial extracellular vesicles (BEVs) into different biofluids, thus becoming a reflection of the microbiota composition in distant sites, and potent biomarkers of disease diagnosis and monitoring [219]. For example, the metagenome of serum-derived EVs showed correlation with bodily microbiota in a murine model of Alzheimer’s disease that differed from the wild type controls [220]; and the presence of *Sphingomonadaceae* in urine-EVs of children with chronic rhinitis and asthma correlated with its upregulation in the airways [221]; and serum bacterial-EVs positively correlated with bacteria in paranasal sinus of patients with rhinosinusitis [222]. In the latter study, microbiota composition varied depending on whether the patients had eosinophilic inflammation or not, thus suggesting a link between microbiota and immune response of the host [222]. Therefore, circulating BEVs as potential biomarkers of dysbiosis in the gut seem promising, since a match between blood EVs and gut microbiota have been repeatedly reported [219]. Interestingly, different bacterial composition and abundances characterize GERD and EoE patients [215,223], suggesting the potential of bacterial-derived EVs in discriminating between these two commonly misdiagnosed diseases.

## 5. Conclusions and Perspectives

Although blood is the source of choice in the majority of studies seeking non-invasive biomarkers, a robust candidate for EoE still has not been found [12]. In this review we show that 18 circulating molecules suggested as disease biomarkers are common for either BA and AD (7), BA and EoE (6) or BA, EoE and AD (5), demonstrating that the co-existence of EoE with other eosinophilic disorders hinders the finding of specific biomarkers. Although our review is limited to concomitancy of EoE with BA and AD, the existence of other common biomarkers for EoE and concomitant allergies such as allergic rhinitis or food allergy is very likely and should be considered for further research. Such findings indicate the need for alternative sampling methods. Other minimally invasive methods employing esophageal mucus seem promising [224,225] and potentially more specific in the context of EoE; however, they are more or less limited to the site of collection. In contrast, blood-based biomarkers have the potential to provide a more complete picture of the state of the disease. To overcome current limitations, further studies should consider either the detailed characterization of patient comorbidities or, when possible, the inclusion of appropriate atopic controls. Additionally, a more refined manipulation of blood samples might improve specificity, such as the enrichment of EVs from patient´s blood. Despite being in its infancy, a considerable number of studies have explored the potential of EVs as biomarkers of disease. In regard to the malignancies covered in this review, BA constitutes the highest number of EV-related studies, doubling those dedicated to AD using blood exclusively. Indeed, up to 19 potential EV-biomarkers were described for BA, five of which had been previously uncovered in circulation including Il-4, IL-5, TNF-α, CCL17/TARC, and miR-21 (Figure 2), thus indicating the specificity and robustness of these biomarkers. In contrast, no coincidences were found between circulating and EV biomarkers described for AD. This is most likely due to the still limited amount of research for this disease. We also noticed a great deal of heterogeneity in the EV-isolation methods employed, as well as an uneven technical accuracy for EV characterization [226,227], highlighting room for improvement, still, within translational research in EVs. Table 2 summarizes all studies involving EVs as potential biomarkers included in this work.

In summary, common circulating biomarkers have been described for BA, AD, and EoE diseases. The suggestion of lack of specificity highlights the need to include an exhaustive control of concomitant atopies. EVs are a promising source that could increase biomarker specificity; however, rigorous characterization and method homogeneity are key to ensuring robust and reproducible results. 

## Figures and Tables

**Table 1:** Research studies including atopic control groups. AEC: absolute eosinophil count, AR: allergic rhinitis, BA: bronchial asthma, AD: atopic dermatitis, AX: food anaphylaxis, CU: contact urticaria, ND: non-determined, EGID: eosinophilic gastrointestinal disorders, EDN: eosinophil-derived neurotoxin, EoP: eosinophil progenitor.

Compared Atopies	Biomarkers Studied	Source of Biomarker	Outcome	Reference
AR, BA, AD, AX, CU, ND	AEC Eosinophil surface marker: anti–IL-5 receptor-α (IL-5Rα) Lymphocyte intracellular cytokines: IFN-γ, TNF-α, IL-4, IL-5, IL-13	Whole blood	− No significant differences of blood eosinophils, IL-5, IL-4, IL-13 between EoE patients, and atopic controls. − Overall, no biomarker was found to differentiate between atopic controls and EoE patients.	[13]
AR, BA, AD, food allergy	3-bromotyrosine (3-BT)	Urine	− Levels of urine 3-BT were significantly increased in EoE patients compared to atopic controls by 13-fold.	[14]
AR, BA	AEC Eosinophil surface markers: CD23, CD54, CRTH2, CD11c, CCR3, CD44, CD11b, CD18, CD58 Cytokines: IL-2, IL-3, IL-5, GM-CSF, CCL5 (RANTES), CCL11 (eotaxin-1), CCL26 (eotaxin-3)	Blood plasma	− Significant increment of AEC in EoE. − Increased CD23^+^, CD54^+^, CRTH2^+^, and CD11c^+^ eosinophils; and decreased CCR3^+^ and CD44^+^ eosinophils in EoE. − Increased blood levels of CCL5 in EoE.	[15]
EGID, eczema, AR, AS, allergic conjunctivitis	AEC Cytokines: IL-5, Eotaxin-1/2/3 Granule protein: EDN	Blood plasma	− No differences in AEC, Eotaxin-3, and EDN level in EoE and atopic controls.	[16]
AR, BA, AD	EoP	Whole blood	− No differences in EoP levels between EoE patients and atopic controls.	[17]
BA, Food allergy	Eosinophil surface markers: CD66b Transcription factor: STAT-1/6	Whole blood	− Increased CD66b+ eosinophils and phosphor-STAT/1 and phosphor-STAT/6 in EoE compared to atopic controls.	[18]
BA	miR-21	Blood serum	− miR-21 is elevated in EoE and asthma patients.	[19]
AR, allergic conjunctivitis, BA, eczema, food allergy	AEC Granule protein: ECP Cytokines: CCL17, CCL18, CCL26 (eotaxin3) Mast cell tryptase (MCT)	Blood serum	− No differences were observed for any biomarker.	[20]

**Table 2:** Studies exploring the potential as biomarkers of circulating EVs included in this work. TEM: transmission electron microscopy, UC: ultracentrifugation, NTA: nanoparticle tracking analysis, BCA: bicinchoninic acid assay, DLS: dynamic light scattering, SEM: scanning electron microscopy, SEC: size exclusion chromatography, PBMC: peripheral blood mononuclear cells.

Disease	Source	Outcome	Isolation Method	EV´s Characterization (According to MISEV ^1^)	References
BA	Plasma	Platelet microparticles (PMPs) are upregulated in asthma (20 asthmatics vs. 15 controls)	Centrifugation (200× *g* and 1500× *g*)	None	[183]
BA	Plasma	Direct correlation of endothelial cell-derived microparticles (MPs) levels and pollution in asthmatics (17 asthmatics vs. 10 controls)	Centrifugation (11,000× *g* and 13,000× *g*)	None	[184]
BA	Plasma	EV-packaged let-7i-5p increased during asthma attacks in childhood asthma (110 asthmatic children)	ExoQuick Plasma Prep with Thrombin kit (SBI, US)	TEM Flow cytometry Western blot	[185]
Type 1 allergy	Plasma	EVs-cytokine cargo discriminates allergic from control patients (22 allergics vs. 16 controls)	Centrifugation (2 h at 110,000× *g*)	BCA NTA Western blot	[186]
BA	Serum	Upregulated miR-126 in EVs of asthmatics (20 allergic asthmatics vs. 16 controls)	Centrifugation (20 min at 2000× *g*, filtered with 0.22 micron-mesh, and 2 h at 110,000× *g*)	EM Western blot	[187]
BA	Serum	Elevated EV-miR-125b serves for diagnostics and assessment of severity in asthma (80 asthma vs. 30 controls)	exoRNeasy Serum/plasma MaxiKit (Qiagen, Germany)	EM NTA	[188]
BA	Plasma	Upregulated EV-miR-125b and downregulated EV-miR-133b, miR-130a and miR-124 in asthmatics (30 asthmatics vs. 30 controls)	Centrifugation (70 min at 100,000× *g*)	DLS TEM Flow cytometry	[189]
BA	Plasma	Significant upregulation of EV-miR-223 and EV-miR-21 in asthmatics (22 asthmatics vs. 24 controls)	Exo-SpinTM kit (Cell Guidance System, UK)	SEM DLS	[190]
BA	Plasma	EV-miR-122-5p is increased in severe asthma (45 eosinophilic asthmatics vs. 16 controls)	SEC (qEV, Izon Science, NZ)	NTA BCA Flow cytometry	[191]
AD	Serum	Specific microbiome-EVs discriminate AD patients (24 atopics vs. 49 controls)	Centrifugation (3000 rpm and 10,000× *g*)	None	[196]
AD	Serum	Distinct subpopulations of T-cell derived EVs can reflect inflammatory status (63 with inflammatory diseases vs. 20 controls)	Total exosome isolation kit (ThermoFisher, Germany)	Fluorescent NTA	[197]
AD	Serum	Increased EV-miR-103a-3p in AD patients (18 atopics vs. 8 controls)	ExoQuick-TC (SBI, US)	TRPS TEM Flow cytometry Western blot	[198]
AD	Plasma	Dysregulation of transfer RNA-derived fragment in EVs in AD (23 atopics vs. 23 controls)	ExoQuick-Plasma prep and Exoome precitation kit (SBI, US)	TEM	[200]
AD	Plasma	Different proteomic profile of EVs in AD (12 atopics vs. 13 controls)	Centrifugation (70 min and 100,000× *g*)	TEM Flow cytometry Western blot	[201]

^1^ Minimal Information for Studies of Extracellular Vesicles (MISEV) is an initiative of the International Society of Extracellular Vesicles (ISEV) that aims to bring agreed criteria to guide scientists in reporting results and distinguish EV from non-EV components.

## Data Availability

Not applicable.
